# *Phaeodactylum tricornutum* as a model organism for testing the membrane penetrability of sulphonamide carbonic anhydrase inhibitors

**DOI:** 10.1080/14756366.2018.1559840

**Published:** 2019-01-27

**Authors:** Alessandra Rogato, Sonia Del Prete, Alessio Nocentini, Vincenzo Carginale, Claudiu T. Supuran, Clemente Capasso

**Affiliations:** a Institute of Bioscience and BioResources, CNR, Naples, Italy;; b Department of Integrative Marine Ecology, Stazione Zoologica Anton Dohrn, Naples, Italy;; c Neurofarba Department, University of Florence, Polo Scientifico, Sesto Fiorentino, Florence, Italy

**Keywords:** Carbonic anhydrase, metalloenzymes, sulphonamide inhibitors, marine diatom, membrane penetrability

## Abstract

Carbonic anhydrases (CAs) are ubiquitous metalloenzymes, which started to be investigated in detail in pathogenic, as well as non-pathogenic species since their pivotal role is to accelerate the physiological CO_2_ hydration/dehydration reaction significantly. Here, we propose the marine unicellular diatom *Phaeodactylum tricornutum* as a model organism for testing the membrane penetrability of CA inhibitors (CAIs). Seven inhibitors belonging to the sulphonamide type and possessing a diverse scaffold have been explored for their *in vitro* inhibition of the whole diatom CAs and the *in vivo* inhibitory effect on the growth of *P. tricornutum*. Interesting, inhibition of growth was observed, *in vivo*, demonstrating that this diatom is a good model for testing the cell wall penetrability of this class of pharmacological agents. Considering that many pathogens are difficult and dangerous to grow in the laboratory, the growth inhibition of *P. tricornutum* with different such CAIs may be subsequently used to design inhibition studies of CAs from pathogenic organisms.

## 1. Introduction

The physiologic/biosynthetic processes requiring CO_2_ or ${\text{HCO}}_{3}^{-}$ (respiration, photosynthesis/gluconeogenesis, lipogenesis, ureagenesis, carboxylation) and biochemical pathways involving pH homeostasis, secretion of electrolytes, calcification, bone resorption, transport of CO_2_, and bicarbonate, etc., are connected with the interconversion of CO_2_ to bicarbonate and protons (CO_2_ + H_2_O ⇌ ${\text{HCO}}_{3}^{-}$ + H^+^)^1^^,^^2^. In all living organism, the CO_2_ hydration/dehydration reaction is catalysed by a superfamily of ubiquitous metalloenzymes, known as carbonic anhydrases (CAs, EC 4.2.1.1)^3–10^, which catalyse these reactions at very high rates, with a pseudo-first order kinetic constant (*k*_cat_) ranging from 10^4^ to 10^6^ s^−1^ for the CO_2_ hydration^11^^,^^12^. At the intracellular concentrations of CO_2_, the uncatalysed CO_2_ hydration/dehydration reaction has a too low rate with an effective *k*_cat_ of 0.15 s^−1^ for the hydration reaction, and a rate constant of 50 s^−1^ for the reverse reaction^11^^,^^12^. CAs have thus the physiologically pivotal role to accelerate the CO_2_ hydration/dehydration reaction significantly, in order to support the fast processes involving dissolved inorganic carbon, which otherwise would be impaired without these enzymes^13^. In this context, CAs started to be investigated in detail in pathogenic, as well as non-pathogenic organisms recently, since it has been demonstrated that CAs are essential for the life cycle of bacteria, fungi and protozoa^4^^,^^9^^,^^13–20^. For example, fungal *β*-CAs plays a critical role in the CO_2_-sensing and in the regulation of sexual development of many fungal organisms^20^. *Plasmodium falciparum,* which is a protozoan, uses its η-CA for producing ${\text{HCO}}_{3}^{-}$, which is the substrate of the first enzyme involved in the pyrimidine pathway and necessary for DNA/RNA synthesis during the exponential growth and replication or the parasite^4^^,^^21^. Again, the growth of two non-pathogenic bacteria *Ralstonia eutropha* (its genome encodes for one periplasmic *ɑ*-CA, two *β*-CAs, and one *ɣ*CA) and *Escherichia coli* (its genome contains one *β*-CA and one *ɣ*-CA) is strictly correlated to the CA activity^22^^,^^23^. The two CAs (*ɑ* and *β*) encoded in the genome of the pathogen *Helicobacter pylori*, are essential for the acid acclimation and the survival of the pathogen within the human stomach^24–26^; *Vibrio cholerae* uses its CAs (*ɑ*, *β* and *ɣ*) for producing sodium bicarbonate, which is an inducer of the cholera toxin gene expression^27–32^. The two pathogenic bacteria, *Brucella suis* and *Mycobacterium tuberculosis*, need functional CAs for growing^33–38^. It is readily apparent that CAs from pathogens are potential drug targets and their inhibition leads to growth impairment or growth defects of the microorganism. Fortunately, many CA inhibitors (CAI) exists, which could be classified as inhibitors binding the metal ion (anion, sulphonamides and their bioisosteres, dithiocarbamates, xanthates); inhibitors anchoring to the water molecule/hydroxide ion coordinated to the metal (phenols, polyamines, thioxocoumarins, sulphocumarins); inhibitors occluding the active site entrance (coumarins and their isosteres) and inhibitors binding out of the active site^39^. As described in the literature, the principal drawback using CAIs as antiinfectives agents is the lack of selectivity towards the pathogenic versus human isoforms^40–43^. For this reason, many research groups are continually involved in the synthesis of new CAIs or in the modification/optimisation of the existing inhibitors, which are commonly tested on CAs from mammalian and pathogenic organisms^11^^,^^13–15^^,^^20^^,^^44–46^. Another critical issue is related to the inhibitor penetrability into microbial cells. Many CAIs are very efficient inhibitors when tested *in vitro* on the purified enzymes (inhibitors with a nanomolar K_I_) but showed ineffective *in vivo* results when tested on the microorganisms^38^^,^^47^^,^^48^. Since it is very complicated to obtain specific control measures and containment levels for activities with pathogenic organisms, in this article, we propose the marine unicellular diatom *Phaeodactylum tricornutum* as a model organism for testing the membrane penetrability of the CAIs. *P. tricornutum* is a eukaryotic organism characterised by fusiform cells with a cell wall poor in silica^49^^,^^50^. The genome of the *P. tricornutum* encodes for nine CAs: five α-CAs confined in the matrices of the four-layered plastid membranes, two β-CAs (PtCA1 and PtCA2) located in the pyrenoid and two mitochondrial γ-CAs^49^. Recently, in the lumen of the pyrenoid-penetrating thylakoid a new class of CAs, named *θ*-CA, has been identified^49^. The CA inhibition of marine diatoms, as well as of other microalgae, lead to growth impairment or defects in the microalga because these enzymes have a pivotal role in ensuring the supply of inorganic carbon (C_i_) to RuBisCO and phosphoenolpyruvate carboxylase^49^^,^^51^.

Here, seven inhibitors belonging to the sulphonamide types, such as the **AAZ**, **MZA**, which are clinically used agents, and compounds **1–5** have been explored for their *in vitro* inhibition of the diatom CAs and *in vivo* inhibitory effect on the growth of the *P. tricornutum* cell. Our results demonstrate that the growth of the *P. tricornutum* cells is affected by the CAIs and the unicellular diatom represents a good model for verifying the CAIs membrane penetrability.

## 2. Material and methods

### 2.1. Chemistry

Compounds **3–5** used in the work were reported earlier by our groups^52^^,^^53^. **AAZ** and **MZA** were commercially available from Sigma-Aldrich (Milan, Italy). All the chemicals and solvents were purchased from Sigma-Aldrich (Milan, Italy). All reactions involving air- or moisture-sensitive compounds were performed under a nitrogen atmosphere using dried glassware and syringes techniques to transfer solutions. Nuclear magnetic resonance (^1^H-NMR, 13C-NMR) spectra were recorded using a Bruker Advance III 400 MHz spectrometer in DMSO-*d_6_*. Chemical shifts are reported in parts per million (ppm) and the coupling constants (J) are expressed in Hertz (Hz). Splitting patterns are designated as follows: s, singlet; d, doublet; t, triplet; q, quadruplet; m, multiplet; bs, broad singlet; dd, double of doubles. The assignment of exchangeable protons was confirmed by the addition of D_2_O. Analytical thin-layer chromatography (TLC) was carried out on Sigma Aldrich silica gel F-254 plates. Flash chromatography purifications were performed on Sigma Aldrich Silica gel 60 (230–400 mesh ASTM) as the stationary phase and ethyl acetate/n-hexane or MeOH/DCM were used as eluents. Melting points (mp) were measured in open capillary tubes with a Gallenkamp MPD350.BM3.5 apparatus and are uncorrected. The solvents used in MS measures were acetone, acetonitrile (Chromasolv grade), purchased from Sigma-Aldrich (Milan-Italy), and mQ water 18 MΩ, obtained from Millipore's Simplicity system (Milan-Italy). The mass spectra were obtained using a Varian 1200 L triple quadrupole system (Palo Alto, CA) equipped by Electrospray Source (ESI) operating in both positive and negative ions. Stock solutions of analytes were prepared in acetone at 1.0 mg mL^−1^ and stored at 4 °C. Working solutions of each analyte were freshly prepared by diluting stock solutions in a mixture of mQ water:acetonitrile 1:1 (*v/v*) up to a concentration of 1.0 µg mL^−1^ The mass spectra of each analyte were acquired by introducing, via syringe pump at 10 µL min^−1^, of the its working solution. Raw-data were collected and processed by Varian Workstation Vers. 6.8 software.

#### 2.1.1. Synthesis of 4-(t-butyl)-N-(3-methyl-5-sulphamoyl-1,3,4-thiadiazol-2(3H)-ylidene)benzamide 1 740


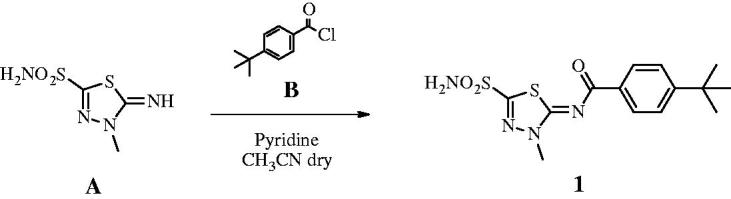


4-t-Butylbenzyl chloride **B** (1.2 eq) was added to a solution of 5-imino-4-methyl-4,5-dihydro-1,3,4-thiadiazole-2-sulphonamide **A** (0.3 g, 1.0 eq) and pyridine (3.0 eq) in dry acetonitrile (5 ml) at 0 °C under a nitrogen atmosphere. The solution was stirred at r.t. until the starting material was consumed (TLC monitoring), then quenched with HCl 1 M (15 ml). The formed precipitate was filtered-off and purified by silica gel column chromatography eluting with 40% EtOAc in n-hexane to afford the title compound **1** as a white solid. 52% yield; m.p. 295–6 °C; silica gel TLC *R_f_*0.47 (EtOAc/n-hexane 60% *v/v*); δ_H_ (400 MHz, DMSO-*d_6_*): 1.32 (s, 9H, 3 × C*H*_3_), 4.08 (s, 3H, NC*H*_3_), 7.56 (d, *J =* 8.8, 2H, Ar–*H*), 8.20 (d, *J =* 8.8, 2H, Ar–*H*), 8.46 (s, 2H, exchange with D_2_O, SO_2_N*H*_2_); δ_C_ (100 MHz, DMSO-*d_6_*): 31.6, 35.8, 39.3, 126.3, 130.1, 133.3, 156.7, 159.0, 166.3, 174.3; *m/z* (ESI negative) 353.0 [M-H]^−^.

#### 2.1.2. Synthesis of t-butyl (2–(4-sulphamoylbenzamido)ethyl)carbamate 2 484


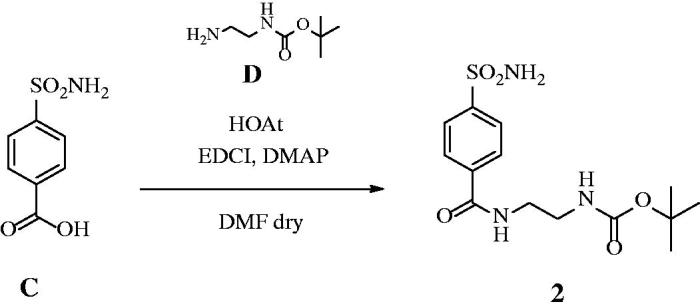


HOAt (1.2 eq) was added to a solution of 4-sulphamoylbenzoic acid **C** (0.2 g, 1.0 eq) and N-boc-ethylenediamine **D** (1.2 eq) in dry DMF (3 ml) under a nitrogen atmosphere, followed by DMAP (0.03 eq) and EDCI (1.2 eq). The solution was stirred at r.t. until the starting material was consumed (TLC monitoring), then quenched with slush (15 ml) and extracted with EtOAc (2 × 20 ml). The organic layers were washed with HCl 0.5 M (2 × 15 ml) and brine (2 × 15 ml), dried over Na_2_SO_4_, filtered-off and concentrated under *vacuo.* The obtained residue was purified by silica gel column chromatography eluting with 10% MeOH in DCM to afford the title compound **2** as a white solid. 73% yield; m.p. 198–199 °C; silica gel TLC *R_f_*0.35 (MeOH/DCM 10% *v/v*); δ_H_ (400 MHz, DMSO-*d_6_*): 1.41 (s, 9H, 3× C*H*_3_), 3.17 (q, *J =* 5.6, 2H, C*H*_2_), 3.34 (q, *J =* 5.6, 2H, C*H*_2_), 6.95 (m, 1H, exchange with D_2_O, CON*H*), 7.50 (s, 2H, exchange with D_2_O, SO_2_N*H*_2_), 7.93 (d, *J =* 8.4, 2H, Ar*–H*), 8.02 (d, *J =* 8.4, 2H, Ar*–H*), 8.66 (m, 1H, exchange with D_2_O, CON*H*); δ_C_ (100 MHz, DMSO-*d_6_*): 29.2, 40.4, 40.4, 78.7, 126.5, 128.8, 138.4, 147.2, 156.7, 166.3; *m/z* (ESI negative) 341.9 [M-H]^−^.

### 2.2. Cell culture

The CCMP632 strain of *P. tricornutum* (Pt1) Bohlin was obtained from the Provasoli-Guillard National Centre for Culture of Marine Phytoplankton. Cultures were grown in f/2-Si medium^54^ at 18 °C under white fluorescent lights (70 μmol m^−1^ s^−1^), 12 h:12 h dark–light cycle as described by De Riso and co-workers^55^. Analyses of the wild-type Pt1 have been performed on cells in exponential phase of growth and collected 4 h after the beginning of the light period.

### 2.3. Spot test analysis

Different dilutions of wild-type Pt1 cells (0.5 × 10^7^; 0.75 × 10^7^; 1 × 10^7^; 1.5 × 10^7^ and 2 × 10^7^ cells) were spotted on f/2-Si agar plates (volume of the spot 5µl). Cells were inoculated with CAIs (**AAZ**, **MZA**, **1–5**) diluted with 10% DMSO at two different concentrations: 0.4 and 1.0 mM. Plates inoculated in an identical manner but only with 10% DMSO and plates inoculated only with f2/-Si were used as control. Cell survival was monitored after 5 days of growth at 18 °C under white fluorescent lights (70 μmol m^–1^ s^–1^), 12 h:12 h dark–light cycle.

### 2.4. Enzyme purification

All the purification steps were carried out at a temperature of 4 °C. Approximately, 10 g of pelleted diatom culture were homogenised in 20 ml of 20 mM Tris-HCl buffer pH 8.3 containing 10^−3 ^M PMSF, 10^−3 ^M benzamidine and 2 × 10^−3 ^M EDTA. The homogenate was centrifuged twice for 30 min at 12,000×*g*, and the resulting supernatant was centrifuged again for 45 min at 100,000×*g*. The hydratase activity was detected in the supernatant fraction. This fraction was dialysed against 20 mM Tris–HCl buffer pH 7.5 and further purified by affinity chromatography on a p-aminomethylbenzenesulphonamide agarose resin (pAMBS; Sigma-Aldrich). 1 ml of pAMBS resin was applied to an empty column (BioRad) and equilibrated with 0.1 M Tris-HCl, pH 7.5 buffer containing 0.2 M K_2_SO_4_, 0.5 mM EDTA. The sample containing about 1 mg of total protein was loaded on the p-AMBS column equilibrated as aforementioned. Unbound proteins were removed by washing extensively with the same buffer. The bound carbonic anhydrase was eluted using 0.4 M KSCN dissolved in 0.1 M Tris–HCl, pH 7.5 buffer. The CA-containing fractions were pooled, dialysed and concentrated by ultrafiltration. The CA-containing sample was subject to SDS-PAGE.

### 2.5. SDS-PAGE

Sodium dodecyl sulphate (SDS)-polyacrylamide gel electrophoresis (PAGE) was carried out according to Laemmli^56^. Samples were dissolved in buffer with 5% b-mercaptoethanol. Gel was stained with Coomassive blue.

### 2.6. Colourimetric carbonic anhydrase assay

CA activity assay was a modification of the procedure described by Capasso et al.^57^. Briefly, the hydratase assay was performed at 0 °C using CO_2_ as substrate following the pH variation due to the catalysed conversion of CO_2_ to bicarbonate. Bromothymol blue was used as pH indicator. The production of hydrogen ions during the CO_2_ hydration reaction lowers the pH of the solution leading to a colour transition of the dye. The time required for the colour change is inversely proportional to the amount of CA present in the sample. The Wilbur-Anderson units (WAU) were calculated according to the following definition: one WAU of CA activity is defined as the ratio (T_0_ − T)/T, where T_0_ (the time needed for the pH indicator colour change for the uncatalysed reaction) and T (the time needed for the pH indicator colour change for the catalysed reaction) are recorded as the time (in seconds) required for the pH to drop from 8.3 to the transition point of the dye (pH 6.8) in a control buffer and the presence of enzyme, respectively.

### 2.7. CA inhibition determination

An Applied Photophysics stopped-flow instrument was used for assaying the total CAs catalysed CO_2_ hydration activity of the algal homogenates. Phenol red was used as pH indicator (0.2 mM) in 20 mM Hepes as buffer (pH 7.5) working at the absorbance maximum of 557 nm or, alternatively, bromothymol blue (0.2 mM in 20 mM Tris, pH 8.3) working at an absorbance maximum of 602 nm. 20 mM Na_2_SO_4_ was added to maintain constant the ionic strength in both media. The initial rates of the CA-catalysed CO_2_ hydration reaction were followed for a period of 10–100 s. For each inhibitor at least six traces of the initial 5–10% of the reaction are used for determining the initial velocity. The uncatalysed rates are determined in the same manner and subtracted from the total observed rates. Stock solutions of inhibitor (0.1 mM) were prepared in distilled-deionised water and dilutions up to 0.01 nM were done thereafter with the assay buffer. The inhibitor and enzyme solutions were pre-incubated together at room temperature prior to assay, in order to allow for the formation of the E-I complex. The rates of the CA-catalysed reaction were followed by measuring the absorbance at the *λ*_max_ of the proper pH indicator, with the inhibition constants obtained by non-linear least-squares methods using PRISM 3 and the Cheng–Prusoff equation as the mean from at least three different determinations.

### 2.8. Lipophilicity determination

Prediction of logP is a method for obtaining information on the partition coefficient of a compound. The platform SwissADME (http://www.swissadme.ch/) was used to calculate the logP of the sulphonamide compounds^58^.

## 3. Results and discussion

### 3.1. Lipophilicity of the sulphonamide CAIs

**Figure 1.**
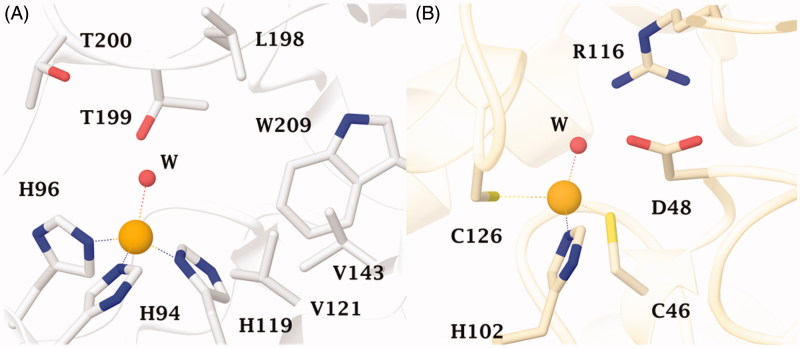
(A) The active site of hCA II as representative of hCA isoforms. The catalytic triad is indicated with the residues: H94, H96 and H119 and (B) The catalytic site of the β-CA from *P. tricornutum* (PtLCIB4**)**. The metal (yellow sphere) is coordinated by two Cys (C46 e C126) and one His (H102) residues and one water molecule (red sphere).

**Figure 2.**
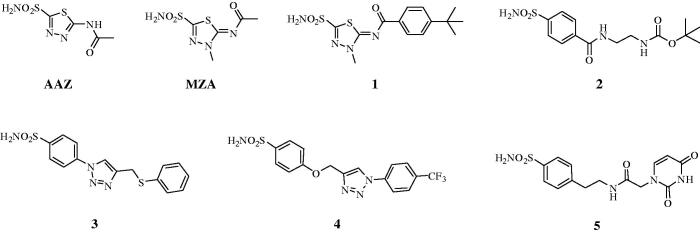
Sulphonamide compounds investigated for their effect on the growth of *P. tricornutum* cells.

Sulphonamides inhibitors with the sulphamoyl group have been extensively studied as CAIs because of their ability to coordinate the metal ions of CA with high affinities (e.g. 10^6^–10^9^ M^−1^ for the human isoform, hCA II)^39^. The zinc is located at the bottom of a deep conical cavity and is coordinated by three histidine ligands (e.g., α-CAs) or two cysteines and one histidine (e.g., β-CAs) and a hydroxide ion with tetrahedral geometry (Figure 1)^39^. The sulphonamide inhibitor nitrogen atom displaces a zinc-bound hydroxide from the active site to form a stable enzyme-inhibitor complex, as elucidated through numerous solved crystallographic structures of various CA-inhibitor adducts^39^. For an optimal *in vivo* activity, a balanced hydrosolubility and liposolubility of the sulphonamide inhibitors are necessary, even if some sulphonamides characterised by low lipid solubility, such as acetazolamide (**AAZ**), are used as effective drugs for a long period. In the present paper, seven sulphonamides inhibitors were investigated for their lipophilicity and effects on the growth of *P. tricornutum*: acetazolamide (**AAZ**), methazolamide (**MZA**), and compounds **1–5** (Figure 2)^52^^,^^53^. The partition coefficient (P) in the system octanol/water, which is one of the essential factors for evaluating the drug penetrability through a biological membrane was calculated by using various commercially available programmes. The seven CAIs showed a lipophilicity consensus ranging from −0.28 to 2.55 (Table 1). A negative consensus LogP value means the compound has a higher affinity for the aqueous phase; when the consensus is 0, the compound is equally partitioned between the lipid and aqueous phases; a positive consensus LogP value denotes a higher concentration in the lipid phase (the compound is more lipophilic). The lipophilicity increases as the consensus LogP values increase (Table 1). From Table 1, it is possible to note that only three of the investigated sulphonamides have low lipid solubility (**AAZ**, **MZA** and **5**), which all incorporate predominantly hydrophilic moieties. The remaining sulphonamides also possess various highly lipophilic moieties (*tert*-Bu-phenyl; phenylthio, T-Bu, trifluromethyl-phenyl, etc) which lead to positive LogP values and thus an enhanced liposolubility over AAZ and MZA (Table 1).

**Table 1.** The lipophilicity of the seven sulphonamides used in the present study.

Compound	Log P_o/w_					
	iLOGP	XLOGP3	WLOGP	MLOGP	SILICOS-IT	Consensus
AAZ	−0.21	−0.26	0.03	−2.75	−0.33	−0.70
MZA	0.05	0.13	−0.34	−1.88	0.02	−0.40
1	2.23	3.46	2.25	0.96	2.13	2.21
2	1.78	0.37	1.67	0.34	−0.12	0.81
3	1.67	1.95	3.14	1.80	0.99	1.91
4	2.45	2.30	4.59	1.88	1.50	2.55
5	0.87	−0.92	−0.38	−0.99	0.01	−0.28

The logP octanol-water partition coefficient (Log *P*_o/w_) value has been obtained using five prediction methods. The consensus is the average of the five predictions methods (iLOGP, XLOP3, WLOGP, MLOGP and SILICOS-IT) and the values are between −0.70 and +2.55.

### 3.2. CAs from P. tricornutum

**Figure 3.**
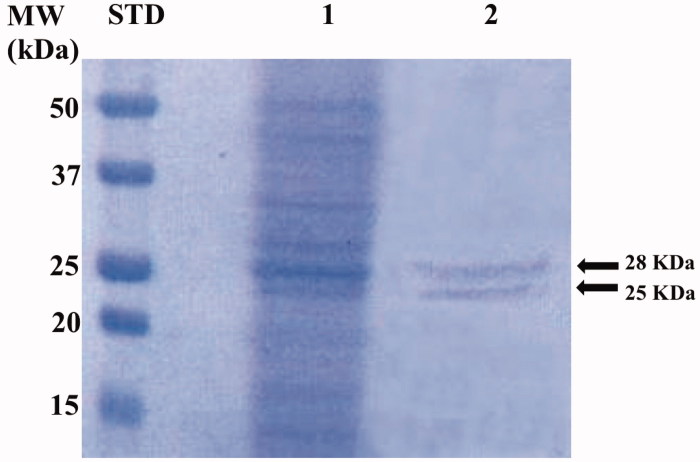
SDS-PAGE of the whole CAs purified by *P. tricornutum* cellular culture. Lane STD, molecular markers, MW starting from the top: 50, 37, 25, 20, and 15 kDa; Lane 1, diatom cell extract; Lane 2, purified whole CAs from the affinity column purification.

The genome of *P. tricornutum* encodes for nine CAs belonging to the α-, β-, γ-, and *θ*-CA classes^49^. The native *P. tricornutum* CAs (PtrCAs) were extracted and purified from 10 g of pelleted diatom culture. Using CO_2_ as a substrate, the activity of PtrCAs in the extract was determined. Most of the CA activity was recovered in the soluble fraction of cellular extract after centrifugation and the calculated specific activity was 200 Wilbur-Anderson units (WAU) mg^−1^ of protein. The extract containing the diatom hydratase activity was further purified by *p*-aminomethylbenzenesulphonamide (pAMBS) affinity chromatography because most of the diatom CA-classes show affinity for this resin as described in the literature^59^. In fact, the resin consists of a cyanogen bromide-activated agarose matrix to which the primary amino group of the pAMBS has been attached. In Figure 3, the SDS-PAGE acquired after the diatom culture homogenisation and column affinity chromatography is shown. The whole native CAs were purified to apparent homogeneity from the other proteins, as indicated by the two single bands evidenced on the SDS-PAGE. The molecular weight estimated by SDS-PAGE was of about 28.0 and 25.0 kDa under reducing conditions. Interesting, the determination of the theoretical molecular weight of the different diatom CA classes, calculated from their amino acid sequences, showed that most of them had a Mw corresponding to 28 and 25 kDa. The diatom CAs were indicated with the acronym PtrCA and are mainly represented by these two bands, as shown by SDS-PAGE (Figure 3).

### 3.3. *In vitro* activity/inhibition of the diatom CAs

The sulphonamide inhibitors described in the previously paragraph, acetazolamide (**AAZ**) and methazolamide (**MZA**), which are clinically used agents, and compounds **1–5**, were used for determining the IC_50_ values relative to the inhibition of the PtrCAs purified from the marine diatom *P. tricornutum* as described above (Table 2). These values were compared with those obtained for the two human CA isoforms (hCA I and hCA II). All the inhibitors considered resulted very efficient versus PtrCAs, with IC_50_ in a range of 8.6–96.6 nM, and the human isoform hCA II (IC_50_ = 1.2–42.1 nM). These inhibitors were less efficient for the human isoform hCA I, and two of them resulted in inefficient inhibitors (IC_50_**1 **=** **1458.9 or IC_50_**4 **>** **10,000). It is interesting to note that among the three CAIs with a low lipophilicity (see Table 1), only the **AAZ** has an IC_50_ of 8.6 nM, which resulted ten times lower respect to those of the two inhibitors with a negative consensus (**MZA** and compound **5**) and the four inhibitors with a good lipophilicity (compounds **1**, **2**, **3** and **4**) (Table 2).

**Table 2.** Inhibition data with the sulphonamides reported here of the two human isoforms, hCA I and hCA II, and the diatom CAs, PtrCAs.

Inhibitor	IC_50_ (nM)^a^		
	hCA I	hCA II	PtrCAs
AAZ	350	21.1	8.6
MZA	97	27.5	92.3
1	1458.9	7.2	85.0
2	375.7	28.7	91.6
3^b^	195.7	1.5	78.6
4^b^	>10,000	15.7	96.7
5^c^	658.9	42.1	86.0

The CO_2_ hydratase assay was determined using the stopped-flow technique.

^a^Mean from 3 different assays, by a stopped flow technique (errors were in the range of ± 5–10% of the reported values).

^b^From Reference ^52^.

^c^From Reference ^53^.

### 3.4. *In vivo* inhibition of growth in the presence of sulphonamides

**Figure 4.**
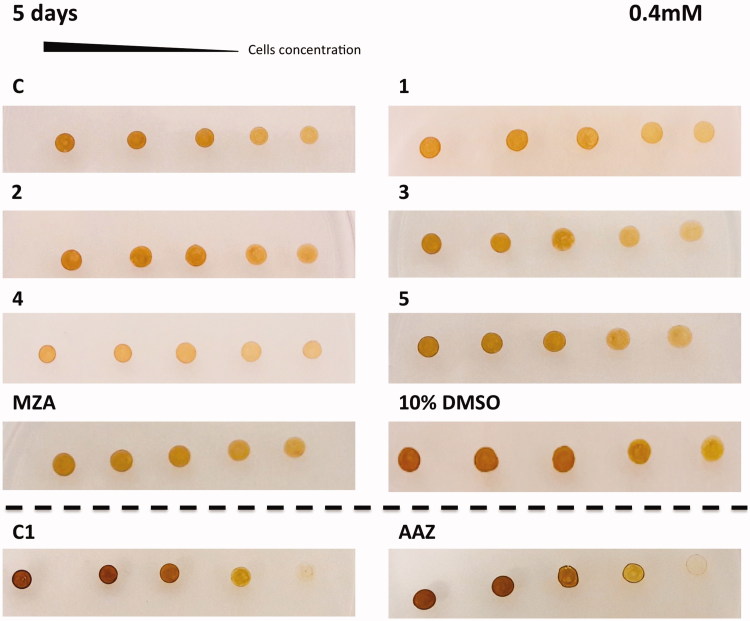
Diatom cells were spotted on the agar plates containing 0.4 mM of each sulphonamide inhibitors. Panel containing the agar with the inhibitor has been indicated with the acronym of the inhibitor used. The panel C1 and AAZ are separated by the other groups because obtained from a different set of experiments. Legend: C and C1, panels with the plate free of the inhibitor; 10% DMSO, panel with the plate inoculated with 10% DMSO; Panels **1**, **2**, **3**, **4**, **5**, **MZA**, **AAZ**, plates with the sulphonamide inhibitors; Number of cells going from left to right: 2.0 × 10^7^; 1.5 × 10^7^; 1.0 × 10^7^; 0.75 × 10^7^ and 0.5 × 10^7^. Diatom cell survival was monitored after five days under normal light conditions and 18 °C. The disappearance of the diatom colonies evidences the inhibitor effect of the CAIs.

**Figure 5.**
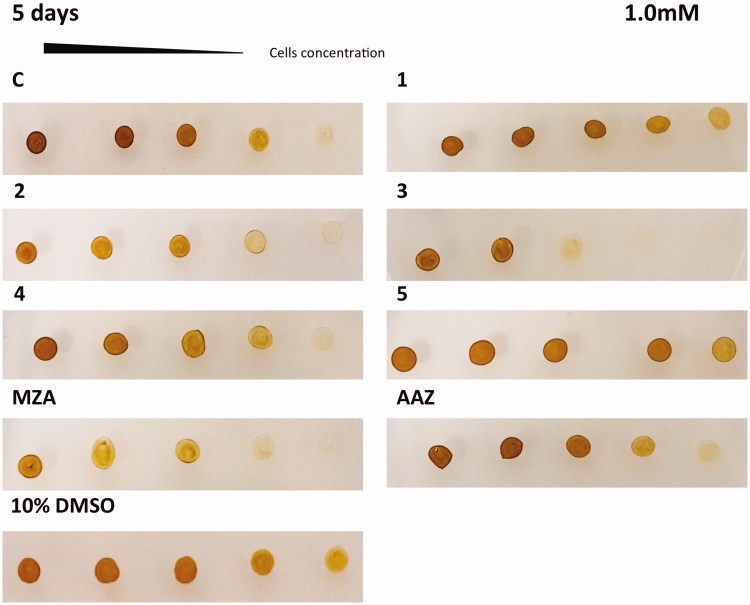
Diatom cells were spotted on the agar plates containing 1.0 mM of each sulphonamide inhibitors. Legend of Figure 5 is the same as Figure 4 except for the **AAZ** panel, which, here, is part of the same set of experiments. The disappearance of the diatom colonies evidences the inhibitor effect of the CAIs.

The *in vivo* cell wall penetrability of the sulphonamide inhibitors **AAZ**, **MZA**, **1–5** cannot be easily quantified, and thus we tested their effects on the cellular growth of *P. tricornutum*. Different numbers of diatom cells were spotted on the agar plates containing two different concentrations of each inhibitor (0.4 and 1 mM) (Figures 4 and 5). The agar plate free of the inhibitor and the plate inoculated with the solvent (10% DMSO) were used as controls. Diatom cell survival was monitored for five days under normal light conditions, at 18 °C. Under laboratory conditions, the effect of the inhibitors on the diatom growth is evidenced by the disappearance of the diatom colonies. As evidenced from Figures 4 and 5, all the sulphonamides CAIs, which were characterised *in vitro* for their ability to inhibit PtrCAs (Table 2), impaired the growth of *P. tricornutum* cells when the plates were supplemented with these sulphonamides at concentrations of 0.4 and 1.0 mM. Compounds **4**, **3**, **1** and **5** showed a noticeable effect on the cellular growth at 0.4 mM concentrations, compared to the controls, and this effect was more pronounced at a low number of cells (Figure 4). At higher concentration of inhibitors (1 mM) it is readily apparent that **MZA**, **2** and **3** resulted to be more effective than the other inhibitors investigated here (Figure 5). However, except for compound **3**, these compounds showed different behaviour when tested at the two different concentrations. For example, **MZA** seems to be not efficient at 0.4 mM, but it interferes efficiently with the diatom growth when used at 1.0 mM (Figures 4 and 5). The stronger effect at a higher concentration of some of these inhibitors may be due to the toxicity problems caused by the off-targeting of other proteins than CAs. Interesting, 10% DMSO, which was the solvent of the inhibitors, had no effects on the cellular diatom growth (Figures 4 and 5). Besides, the results obtained using a concentration of inhibitors of 0.4 mM reflect those of the lipophilicity. **AAZ**, **MZA** and **5**, which have low lipophilicity, were the less effective inhibitors of the diatom cell growth because of difficulties to penetrate through the membrane. **AAZ** has a LogP of −0.70 and is an excellent *in vitro* inhibitor when tested on the whole diatom CAs. This inhibitor failed to show growth impairment of the cells when used at 0.4 mM, while **MZA** and **5**, with lipophilicity values higher than **AAZ**, showed a better inhibition of growth at 0.4 mM (Figure 4).

## 4. Conclusions

In this study, we proposed the marine unicellular diatom *Phaeodactylum tricornutum* as a model organism for testing the membrane penetrability of CAIs and their effects on the growth of the organism. *P. tricornutum* is an eukaryotic organism characterised by fusiform cells with a cell wall poor in silica^49^^,^^50^. The scarcity of silica in the composition of the cell wall makes possible to consider these diatoms as a good model for testing the membrane penetrability of small molecules, such as the CA sulphonamide inhibitors. The seven sulphonamides inhibitors **AAZ, MZA**, and compounds **1–5**, resulted to be effective inhibitors of the diatom CAs when tested *in vitro* (IC_50_–s in the range of 8.6–96 nM). Some of these inhibitors probably can cross the membrane of *P. tricornutum* possessing both hydrophobic and hydrophilic moieties in their molecule. The most efficient *in vivo* inhibitors were compounds **4, 3, 1** and **5** with a noticeable inhibitory growth effect at a low number of cells. Furthermore, these sulphonamides inhibitors showed a different behaviour when tested at the concentrations of 0.4 and 1.0 mM, which is difficult to rationalise. For example, **MZA** was not efficient at 0.4 mM, but it interfered efficiently with the diatom growth when used at 1.0 mM. Probably this effect might be due to the off-targeting of other proteins than the CAs considered here. In general, these results demonstrate that *P. tricornutum* might be considered as an excellent and rather simple model for testing the membrane penetrability of new CAIs and their effects on the growth of the organism. Considering that many pathogens are difficult and dangerous to grow in the laboratory, the preliminary results obtained for the growth inhibition of *P. tricornutum* with different such CAIs may be subsequently used to design inhibition studies of CAs from pathogenic organisms, which may pave the way to novel anti-infectives with a diverse mechanism of action.
